# Building capacity in health systems with and for caregivers: A photovoice protocol

**DOI:** 10.1371/journal.pone.0358550

**Published:** 2026-09-15

**Authors:** Kristina Fuentes, Danielle Jacobson, Arija Birze, Lauren Cadel, Dianne Fierheller, Michelle Marcinow, Bhavini Patel, Abby Vijayakumar, Ian Zenlea, Kerry Kuluski, Elizabeth Mansfield

**Affiliations:** 1 Institute for Better Health, Trillium Health Partners, Mississauga, Ontario, Canada; 2 Institute of Health Policy, Management, and Evaluation, University of Toronto, Toronto, Ontario, Canada; 3 Leslie Dan Faculty of Pharmacy, University of Toronto, Toronto, Ontario, Canada; 4 Dalla Lana School of Public Health, University of Toronto, Toronto, Ontario, Canada; 5 Temerty Faculty of Medicine, Department of Paediatrics, University of Toronto, Toronto, Ontario, Canada; 6 Department of Occupational Science and Occupational Therapy, University of Toronto, Toronto, Ontario, Canada; University at Buffalo School of Medicine and Biomedical Sciences: University at Buffalo Jacobs School of Medicine and Biomedical Sciences, UNITED STATES OF AMERICA

## Abstract

**Introduction:**

Unpaid caregivers—those who provide essential support to family, friends, or neighbours with disabilities, chronic conditions, mental health, or other health concerns—play a critical role in sustaining Canada’s health and social systems. Despite their significant contributions, caregivers remain undervalued, under-recognized, and under-supported. This marginalization has led to elevated rates of burnout, mental health distress, and financial strain, disproportionately impacting women and racialized populations. The COVID-19 pandemic further revealed the systemic risks and potential harms of excluding caregivers from care environments (e.g., hospitals, long-term care settings), prompting urgent calls for reform. This Photovoice study forms part of a broader four-year research program engaging caregivers from diverse communities to co-develop evidence and advocacy tools to support best practices in healthcare for engaging caregivers and inform a new Research and Innovation Hub focused on caregiving.

**Methods:**

Using a community-based participatory research (CBPR) approach, a series of Photovoice workshops will be conducted with four priority caregiver groups in Peel Region, Ontario: (1) caregivers of children with complex care needs; (2) caregivers of seniors; (3) caregivers supporting young adults with mental health challenges; and (4) women caregivers supporting multiple generations. Each group will participate in six virtual workshops focused on photo taking, narrative development, collaborative theming, and advocacy planning. The study foregrounds caregivers as co-researchers, not just participants, and integrates inclusive research practices including compensation, ongoing consent, check-ins, and other supports throughout the process. Data will be collaboratively analyzed with participants to identify cross-cutting themes and policy and practice recommendations. A follow-up qualitative evaluation will assess co-researcher participants’ experiences with the method and its impact.

**Discussion and conclusion:**

This study centers caregiver perspectives, experience, and expertise to inform responsive health system change. Employing CBPR and Photovoice principles will help to amplify caregiver voices and generate actionable knowledge aimed at addressing structural inequities in caregiving policy and practice.

## Introduction

Caregivers play a critical, yet under-recognized and under-supported role in Canada’s healthcare system [1]. In the context of the proposed research, the term “caregivers” is defined as those who provide unpaid care and support for family, friends, or neighbours with disabilities, medical conditions, or mental wellness concerns. Other terms used to refer to this group include “informal caregivers”, “unpaid caregivers”, or “carers” [2]. Research indicates that in Canada, one in every four individuals engages in caregiving, cumulatively investing over five billion hours annually in this role [3]. Further, caregivers contribute billions of dollars every year to Canada’s health and social system by taking on unpaid duties that would typically be provided through paid services [4].

The specific activities undertaken by caregivers vary depending on the type and extent of support required by the person being cared for. Overall examples of such activities include personal and household care (e.g., bathing, feeding, dressing, cleaning, laundry and other housekeeping), medical and health-related tasks (e.g., managing medications, attending medical appointments), emotional and social support, and advocacy [5]. Looking at particular examples, caregivers of individuals with cancer have been reported to assist with treatment-related decisions [6], mental health support [7], scheduling appointments, providing transportation, care coordination [8], personal care, translation, portering, advocacy, and ensuring patient inclusion in care [9]. Similarly, caregivers of older adults assist with “hands-on” care [9], personal care, instrumental activities of daily living support (e.g., cooking, laundry), and conveying patient-related needs and information to healthcare providers [5].

Caregivers are therefore integral actors in health and social care systems who must be recognized, included, and supported in their roles [5,10,11]. In fact, caregiver presence has been associated with improved patient experiences, increased patient safety [12], and decreased complications [13]. Concordantly, their absence is associated with significantly poorer patient outcomes and experiences as well as with increased strain on formal healthcare resources [14,15]. Caregivers’ vital role in healthcare system operations was only further substantiated by the circumstances brought about by the COVID-19 pandemic. For example, during the height of the pandemic, the “no visitors” policy implemented across Canadian hospitals meant that caregivers of patients receiving treatment could not contribute their vital work on healthcare teams [9]. The work caregivers could no longer contribute left gaps in care, which were transferred to overburdened and under-resourced healthcare providers who often were unable to fulfill these extra care-related tasks. As a result, patient safety, patient experiences, and care quality declined [9]. Thus, perhaps unsurprisingly, the Canadian Centre for Caregiving Excellence [16] states that “if all caregivers took a week off, every Canadian would experience the collapse of our care systems before noon on the first day” (p. 1).

Despite the significant contributions made by Canada’s caregivers, there is a noted lack of support and resources available [5,17]. The negative impacts of the under-valuing and under-resourcing of caregiving are increasingly evident. For example, results from the 2023 National Caregiving Survey highlighted that one in four caregivers report fair or poor mental health and that 37% experienced financial hardship due to their caregiving role [1]; in another national survey (3000 + caregivers), it was 63% [17]. Financial strain for caregivers has been noted in other work due to the caregiving commitment, including time off work (and a lack of work-related flexibility and accommodations), resources required to support care that are paid out of pocket (e.g., extra homecare, accessibility-related equipment, incontinence products), and difficulty attaining government supports (e.g., due to restricted eligibility, lacking funds, and cumbersome application processes) [5,17].

Given the personal strain and lack of resources experienced by caregivers, especially in the context of the ongoing health system recovery from the COVID-19 pandemic, caregiver stress and burnout have further intensified. For example, the Canadian Institute for Health Information (CIHI) [18] reports that one in three caregivers of people receiving homecare are distressed (i.e., “the inability to continue with caring activities”, p. 1). While respite services are known to prevent burnout [19], just 8% of caregivers surveyed by the Canadian Centre for Caregiving Excellence (CCCE) report access to such services [17]. Inadequate structural support for caregivers not only threatens their well-being but also has broader implications for health system sustainability, economic productivity (e.g., through workforce attrition and out-of-pocket care costs), and gender equity (given that caregiving responsibilities disproportionately fall on women) [20,21]. To address this gap, there is a critical need to identify, implement, and evaluate evidence-informed practices that support caregivers. This includes amplifying caregiver voices, deepening understanding of the financial, emotional, and physical tolls of caregiving, and co-designing responsive policy and service solutions in partnership with caregivers themselves.

## Materials and methods

### Study objectives

The Photovoice study outlined in this protocol is a foundational component of a broader four-year research program being conducted in collaboration with the CCCE. The broader program aims to advance caregiver-informed health-system priorities and establish a Research and Innovation Hub focused on caregivers’ essential role in health and social systems. The overarching objectives of the broader program are to: 1) co-design policy and practice recommendations by understanding the lived experiences (and user-centred priorities) of caregivers from diverse communities and health contexts; 2) mobilize evidence and build lived expertise knowledge to help communicate and amplify caregiver voices for policy, system, and practice change; 3) co-design an implementation toolkit to support health and social systems in adopting best practices for caregivers; and 4) establish a Research and Innovation Hub focused on caregivers’ essential role in health and social systems.

This protocol specifically outlines plans for a Photovoice workshop series and related knowledge translation activities. Photovoice is an inclusive and participatory arts-informed method [22–24] that allows for lived experience to be explored, better understood, and leveraged in advocacy work. The research team will work alongside caregivers from diverse communities to co-create a Photovoice gallery and collaborative themed analysis that highlights promising practices, challenges, and priorities for advocacy and systems change [22,23]. The evidence, recommendations, and knowledge translation outputs generated through the Photovoice activities will contribute to the overarching objectives of the broader research program.

### Study design

Photovoice is an arts-informed method that involves co-researchers engaging in a series of collaborative group workshops where they take photographs or select images in response to a guiding research topic directed toward bringing about social change [23]. Keeping with best practices in CBPR approaches, the term “co-researchers” is used in this protocol to describe what are traditionally called “participants”. The term “co-researchers” communicates the participatory and equitable roles that caregivers play throughout this project and their involvement in the planning and carrying out of research tasks [25].

The Photovoice method has often been used when working alongside individuals and communities whose perspectives have historically been neglected in service and program delivery [24]. The flexible and inclusive approach to knowledge sharing and communication (i.e., providing opportunities for visual rather than just text-based narratives) makes Photovoice a promising method for gathering information about contextualized experiences [24,26,27]. Another key strength of this approach is the capacity to capture local knowledge and information through the perspectives of community members and raise situated awareness about the need for policy, practice, and systems changes [22]. Photovoice has participatory underpinnings and an advocacy and action-oriented approach [28], which makes the method well-suited for contributing to this project’s objectives of amplifying caregiver voices and developing an implementation toolkit to support health and social systems in adopting best practices for caregivers.

***A community-based participatory research approach:*** In alignment with Community Based Participatory Research (CBPR) approaches, the Photovoice method encourages self-expression, creates opportunities for advocacy and learning in supportive group settings, and can help make traditionally hierarchical research spaces more inclusive [22]. A key tenet of CBPR is the creation of a collaborative, equitable, and supportive research and knowledge dissemination environment where community members with lived experience contribute their skills and expertise and participate in decision-making processes [29–31]. A CBPR approach requires meaningful community involvement and a flexible, iterative process that integrates academic and community-based knowledge throughout the research cycle [29,31,32]. Best practices will be followed for public engagement in healthcare such as significant contributions to research activities and training, recognition and inclusion in publications (including as co-authors) and other knowledge dissemination products, appropriate compensation through stipends, and equity between all members of the research team [33–35].

Creating a Community Advisory Board (CAB) is a key component of CBPR and supports community engagement and local involvement in shaping research and knowledge translation agendas [36]. In September of 2024, a CAB was formed comprising current and former caregivers with experience supporting children, parents, spouses, friends, and older adults across a range of health and social care contexts. Members also bring experience in caregiver and patient advisory roles, community services, disability advocacy, home care, seniors’ services, research, and health-system leadership. Their lived, community, and professional experiences are grounded in the Peel Region context, enabling them to provide locally relevant guidance throughout the Photovoice project. The purpose of this group is to support and inform the development of all phases of the photovoice project described in this protocol. To support CAB sustainability, members receive an annual stipend for their advisory work and commit to a one-year term. CAB members also are provided with an option to renew at the end of each year over the anticipated four-year project period. Under the leadership of the principal investigator (KK) and a community co-chair (BP), members of the CAB will provide guidance throughout the photovoice project research cycle, including the provision of feedback on recruitment strategies and knowledge translation activities.

### Study setting

Trillium Health Partners is a large community and academic teaching hospital that engages a rapid-learning health system model and is located in the Peel Region, Ontario [37]. Peel Region is one of the most diverse communities in Canada with more than 50% of residents having immigrated to Canada [38]. In 2021, approximately 69% of Peel residents belonged to racialized population groups group, with South Asian residents comprising the largest racialized population in the region, followed by Black, Filipino, Chinese, and Arab residents [39,40]. Recent immigrants—defined as immigrants who first obtained landed immigrant or permanent resident status between January 1, 2016 and May 11, 2021—comprised approximately 14.0% of Peel’s immigrant population [40,41].These demographic characteristics highlight this setting’s potential to learn from caregivers from diverse communities to improve the knowledge base informing local and national caregiver strategies. Although maximum variation sampling [42] will be used to recruit eligible caregivers with a range of identities and experiences, the final sample composition will depend on who expresses interest and is able to participate, and therefore may not proportionally reflect Peel Region’s population distribution.

### Ethics approval

This study received approval from the Trillium Health Partners Research Ethics Board (REB ID: #1258).

### Study participants

The Photovoice activities will involve working alongside four separate groups of caregivers: 1) caregivers of children with complex care needs; 2) caregivers of seniors living with a disability, medical condition, or illness; 3) caregivers supporting young adults with mental health challenges 4) women caregivers supporting multiple generations (e.g., both young children and aging parents, or caring for aging parents, adult children, and young grandchildren–all of which, in this study context, are referred to as the ‘Sandwich Generation’). These diverse caregiver populations were selected in alignment with the strategic priorities of the organization where this study is based, which include a commitment to supporting the health, wellness, and program development for people across the lifespan.

To participate in this study, caregivers must meet the following eligibility criteria**:** 1) reside in Peel Region; and 2) be over the age of 16. There is a preference for co-researchers to be comfortable conversing in English due to the complexity of organizing translation activities for Photovoice, which relies on group engagement and collaboration. Translation will be facilitated by an interpreter if a pre-established group arises who prefers to conduct the workshop series in another language. If one individual requests translation support for a workshop series facilitated in English, the research team will strive to accommodate this. Additional eligibility criteria for each group are outlined below. Any criteria may be refined based on discussion with the CAB, project team members, and broader community member input.

**Group 1 criteria** (***caregivers of children with complex care needs):*** Have experience as an unpaid parent, family member, friend, or other support for a child aged 0–18 years with a diagnosed or suspected long-term developmental or behavioural condition, such as autism spectrum disorder, developmental delay, or behavioural challenges that affect daily activities. The child must experience noticeable challenges in everyday functioning compared with peers of a similar age, require ongoing healthcare, therapeutic, community, or school-based services or supports (whether or not these are currently available), and require a level of care coordination or advocacy that places significant demands on the family.

**Group 2 criteria** (***caregivers of seniors)*:** Have experience directly caring for a friend or family member who is 65 years of age or older.

**Group 3 criteria (*caregivers supporting young adults with mental health challenges):*** Have experience supporting a friend or family member seeking or receiving mental health services.

**Group 4 criteria** (***caregivers supporting multiple generations)****:* 1) self-identify as a woman; 2) be a caregiver for at least two generations simultaneously, with at least one of those individuals having a disability, medical condition, or illness.

### Sampling and recruitment

The sampling approach will be purposive and will use maximum variation sampling to support the inclusion of community members representing a diverse range of identities (i.e., age, gender, racialized group, ethnicity, religion, education) and experiences, rather than to achieve demographic representativeness. There will be a multi-faceted approach to recruitment involving ongoing collaboration with community partners serving caregivers in Peel Region as well as with CAB members. All research processes will be guided by best practices in Equity, Diversity, and Inclusion research [43] to ensure the representation and inclusion of diverse experiences and identities of caregivers.

During an initial telephone screening conversation, the researchers will assess eligibility and co-researcher attributes to support a maximum variation sampling approach, which involves purposively seeking co-researchers with a range of identities and caregiving experiences to capture diverse perspectives within each workshop series. Approximately 8–10 co-researchers will be recruited for each workshop series, with an expectation that the final number of co-researchers will be between 6–8 per group, based on typical study attrition. Informed by previous online Photovoice studies and a recent literature review conducted by members of the project team [44], 6–8 co-researchers is an optimal number for ensuring diversity of experience and meaningful engagement in the Photovoice workshops.

Electronic flyers will be distributed to collaborators for dissemination through email distribution lists and posting at recommended sites and on partners’ social media platforms. These collaborators may include caregiver support and advocacy organizations, community health and social service organizations, hospital-based programs and clinics, patient and family advisory groups, and organizations serving children with complex care needs, older adults, young adults with mental health challenges, and women in multigenerational caregiving roles (i.e., sandwich generation caregivers). Organizations interested in learning more about the project will contact members of the research team who will then explain the project and how they can get involved. Project team members with links to additional organizations serving caregiver groups in Peel will send out a communication through their social media including LinkedIn, Facebook, and Instagram. Finally, members of the project team may attend events or group activities where eligible participants may be in attendance, to present the project.

Individuals expressing interest in learning more about or participating in the project will contact the project team directly. A member of the research team will then respond to potential co-researchers via email to provide an introduction overview of the project and schedule a meeting via Zoom or by phone (based on preference). This meeting will involve an initial conversation about the project and eligibility assessment to support the maximum variation sampling approach. Project applicants who do not meet eligibility criteria will be sent a thank you note. Individuals who meet project eligibility criteria will be provided (via email) a project information package consisting of details about the project, what participation entails, and informed consent forms and processes. The researcher will then schedule a second call with eligible individuals to review the information package, ensuring ample opportunity for questions. Verbal consent will be documented during this call and stored separately from co-researcher data. Co-researchers will also be given the option to provide written consent if they would prefer to do so.

### Data collection and analysis

#### Phase 1: Photovoice workshops.

A separate Photovoice workshop series will be held with each of the four distinct groups of caregivers (i.e., caregivers of: 1. children with complex care needs, 2. seniors, 3. individuals with mental wellness concerns, 4. multiple generations). The method of holding separate workshops for the different groups has been successfully implemented by project team members in previous Photovoice projects and is intended to support an open discussion environment and nuanced, contextualized understandings of distinct caregiver populations. Each of the four Photovoice workshop series will consist of six workshops, two-hours in duration, with a two-week time period in between each workshop. The four workshop series will be conducted sequentially over approximately twelve months. Data collection and collaborative theming will be completed for each group before the findings are brought together through the planned cross-group knowledge translation activities. Drawing from best practices identified through previous arts-informed work and a recent scoping review published by team members [44], three members of the research team will be involved in co-facilitating each workshop series (all of whom have been trained in the Photovoice method). Two co-facilitators will lead the discussion while a third facilitator will provide technical support (e.g., admitting co-researchers into the Zoom room, sharing the workshop slides, monitoring the chat, providing support for co-researchers experiencing difficulty connecting or sharing their screen, etc.).

The Photovoice workshops will be held virtually, over Zoom, unless co-researchers request that they be held in-person. Traditionally, Photovoice workshops have been primarily held in-person. However, in recent years, and largely in response to safety and logistical concerns raised by the COVID-19 pandemic, Photovoice projects are increasingly conducted online or in hybrid formats, with a mixture of online and in-person activities [44]. Virtual and hybrid Photovoice projects have many benefits when compared with strictly in-person methods, as they facilitate greater comfort for some individuals, allow for scheduling flexibility, and reduce transportation costs/time as well as barriers to childcare [44–46].

Co-researchers will be encouraged to take photos in their living spaces and public places that focus on objects, scenery, metaphorical images, and artwork (e.g., drawings, cartoons, poetry), rather than images of identifiable individuals. Alternatively, co-researchers may access online images that are clearly identified as being in the public domain or otherwise licensed for reuse. This option may provide a lower-barrier starting point for co-researchers who are initially hesitant to take or share personal photographs, including when reflecting on sensitive or stigmatized experiences. The research team will provide guidance on locating appropriate images and documenting their source and licensing information. Any image for which reuse permissions cannot be verified will not be included in public-facing knowledge translation materials; co-researchers will instead be supported to replace it with an original photograph or artwork. Each of the four co-researcher groups will be asked to co-create and refine an experiential prompt and/or a future-oriented prompt such as: 1) Experiential prompt: *What images reflect your experiences and perspectives on caregiving?* 2) Future oriented prompt: *What does a society that values and supports your role as a caregiver look like?* Prompts will be tailored according to the focus of each group. Co-researchers will then be asked to take photos or choose images in response to the co-created prompt.

The four phases of Photovoice are based upon the methodological principles outlined by Wang & Burris [23]. The workshop session alignment with the phases and the agenda of activities for each meeting is outlined in Table 1. The number and timing of workshops may vary slightly for each workshop series depending on each group’s preference.

**Table 1:** Overview of Photovoice Workshops.

Workshops	Agenda & Activities	Co-researcher Take home activity
*Pre-workshop onboarding: Prior to each workshop series, project team members will schedule a call/meeting over Zoom with each co-researcher (i.e., participant) who has consented to join the workshop to review the project information package, Zoom technology, and remote meeting best practices (e.g. wearing headphones, quiet workspace).*		
**1. Introduction to the project and Photovoice**	• Ice breaker and introductions • Review of project purpose and Photovoice methods • Review of research ethics, privacy, confidentiality, and best practices	**• Assignment for workshop 2:** Bring a picture of a hobby, interest, or passion that says something about who you are and that you are comfortable sharing in a group setting
*There will be a minimum of one week between Workshops 1 and 2.*		
**2. Co-designing the photo prompt and Photovoice assignment preparation**	• Ice-breaker activity and rounding • Co-designing project prompts • Instructions for Photovoice assignment (e.g. photo-taking tips, symbolic/metaphorical vs literal images, selecting images, and file upload instructions) • Review of Photovoice ethics including guidelines/rules around photo-taking and ownership and use of images	**• Assignment for workshop 3:** Take or select two to three images or photos in response to the Photovoice prompt
*During the two-week time period between workshops 2 & 3, project team members trained in Photovoice will check in with co-researchers about the Photovoice assignment. In addition, two drop-in ‘office hours’ (1 hour each) will be held (early afternoon, evening option) for those who would like to discuss the project and or assignment further.*		
3. Photo sharing and caption development	• Ice-breaker activity and rounding • Select co-researchers’ top 2 photos and work in small groups (breakout rooms in Zoom) to discuss images and captions • Explain meaning of images in relation to the research question • Present Photovoice narratives with the group	**• Assignment for workshop 4:** Complete a working caption for two to three photos that you would like to share in workshop 4 ◦ Option to take or select two to three additional photos in response to the prompt
*During the two-week time period between workshops 3 & 4, project team members trained in Photovoice will check in with co-researchers about the Photovoice assignment and provide support for caption development and refinement. In addition, two drop-in office hours (one hour each) will be held (early afternoon, evening option) for those who would like to discuss the project and or assignment further.*		
4. Caption refinement	• Ice-breaker activity and rounding • Focused small group work to share and provide feedback on photos with captions • Theming practice exercises with open-source photos • Focused small group and large group discussions to identify cross-cutting Photovoice topics and clusters about caregiving experience and improvement recommendations	**• Assignment for workshop 5:** Select top three photos with finalized caption for possible inclusion in exhibit and/or e-zine (an electronic magazine format that typically includes images and texts)
*During the two-week time period between workshops 4 & 5, project team members trained in Photovoice will check in with co-researchers about support for Photovoice narratives. Two optional drop-in ‘office hours’ (1 hour each) will be held (early afternoon, evening option) with project team members to introduce e-zine format and e-zine examples for a virtual exhibit of the Photovoice narratives.*		
5. Group theming for Photovoice	• Ice-breaker activity and rounding • Continuing from previous workshop: Focused small group and large group discussions to identify cross-cutting Photovoice themes	**• Assignment for Workshop 6:** Review group notes describing how the Photovoice narratives could be themed/organized and reflect upon advocacy and local change messages
*During the two-week time period between workshops 5 & 6, project team members trained in Photovoice will check in with co-researchers about the completed Photovoice narratives and discuss consent for sharing the Photovoice narratives in exhibit and e-zine (electronic magazine) formats. Two optional drop-in office hours (1 hour each) will be held (early afternoon, evening option) with project team members to discuss content for a themed e-zine format for a virtual exhibit of the Photovoice narratives.*		
6. Group theming and Photovoice exhibit co-design planning	• Ice-breaker activity and rounding • Focused small group and large group discussions to identify cross-cutting Photovoice themes • Discuss and plan for Photovoice exhibit (choose which themes to include in exhibit and review formatting options such as background colours for photos, etc.)	**• Photovoice sharing consent discussion:** Consent discussion with team members about potential inclusion of Photovoice narratives in Photovoice exhibit and e-zine formats
*Two weeks following the final workshop meeting, co-researchers will be provided with a thank you letter, a certificate of Photovoice workshop completion and a personal digital project photo album.*		

The project team will engage in the following synchronous and asynchronous activities throughout the course of the Photovoice work:

1. ***Project team debriefing:*** Immediately following each online Photovoice workshop, the co-facilitators will meet via Zoom to reflect on how the session went, discuss technical or other issues that may have arisen and related actions that need to be taken, and identify next steps, including any follow-up communications to be sent to co-researchers.
2. ***Ongoing project team communications with co-researchers*:** Throughout each workshop series, the three facilitators leading the workshop will engage in ongoing communication with co-researchers through weekly group email updates as well as one-on-one email check-ins. This will ensure that co-researchers remain engaged and feel supported throughout the Photovoice process, which is particularly important given that this method involves substantial ‘homework’ in between workshops (e.g., taking photos, revising captions, etc.).
3. ***Virtual office hours*:** Virtual ‘office hours’ will be held every two weeks while each workshop series is running, to provide synchronous opportunities for co-researchers to ask questions or seek additional support for the Photovoice activities. Additionally, Photovoice co-facilitators will be available to meet with co-researchers via Zoom or phone and to provide support by email.

The Photovoice analysis will be driven by the CBPR commitment to valuing and prioritizing co-researcher lived experiences and expertise [47]. The project team will engage co-researchers in participatory data analysis through collaborative theming, an iterative process which will take place over the course of the final three workshops in each workshop series. Each group will engage in collaborative theming discussions. Given the small, purposively selected groups, the analysis will not be designed to make systematic comparisons between ethnocultural groups or to generalize particular themes to these communities, although co-researchers may choose to explore similarities and differences across experiences if they arise organically during collaborative analysis. Following this, a themed slide deck—in which photos and captions are organized and grouped according to the collaboratively identified themes—will be shared with co-researchers for further feedback and refinement. The findings will be shared with the CAB members for feedback and discussion, and co-researchers from relevant groups will have the opportunity to attend.

#### Phase 2: Photovoice evaluation.

Following the completion of the four Photovoice workshop series, one for each caregiver group and each consisting of approximately six workshops (see Table 1), co-researchers across all groups (up to 20 total, or 5 from each group) will be invited to participate in feedback interviews or focus groups. The purpose of these sessions will be to assess co-researchers’ experiences during these activities and to explore the strengths and limitations of using this participatory visual method as it relates to helping achieve the study objectives. The research team has drawn from Graham Gibb’s reflection cycle [48] to develop questions that will be asked about co-researcher perspectives on what worked, what did not work, and how the process could be improved for future online Photovoice initiatives (see S1 File).

The interviews and focus groups will be held online (via Zoom), will be conducted by a trained qualitative researcher on the project team, and will be audio-recorded, with co-researcher consent. The audio recording will be professionally transcribed, reviewed for accuracy, de-identified, and entered into NVivo, a qualitative software management program.

***Feedback evaluation data:*** Thematic analysis will be used to identify themes across the feedback evaluation datasets [49,50]. The research team will review transcripts and then will meet to compare independent coding notes and develop a coding framework. Two research team members will then code the data using this coding framework. All data will be double coded by two members of the research team who will meet with the Principal Investigators (KK and EM) to discuss and resolve differences in interpretation and to ensure consistency in coding usage. Codes will be combined into themes during a series of team meetings in which key findings will be explored in relation to the research objective of evaluating the strengths and challenges with the arts-informed work.

### Data management and privacy

Several strategies will be employed to ensure co-researchers’ privacy and confidentiality, which have been utilized successfully in previous online Photovoice projects led by EM. Any information pertaining to co-researchers in this study will be kept private and confidential. Each co-researcher will be assigned a unique identifier and pseudonym. Co-researchers’ audio recorded consent, names, and contact information will be stored separately from their data on a password-protected, secure drive with access limited to only the project team. Raw data from the audiotaped meetings and workshops will be encrypted and password protected. Any names of individuals, organizations, locations, or other identifying details will be removed from the transcribed audio-recordings, which will be checked for quality. All relevant study documents (e.g., names, contact information, raw data, audio recordings, transcripts, electronic files) will be destroyed seven years after the completion of the study. No personal identifying information will be included in any reports, presentations, or publications arising from this research, and co-researchers’ pseudonyms will be used in all relevant communication materials.

Photographs and annotated narratives will be assigned unique identifiers to which only the project team will have access. This list will be stored on a secure server that is separate from the data. The sharing of specific photographs/narratives during group Photovoice activities will only take place with the co-researcher’s consent. To facilitate collating photo images collected through the Photovoice activities, the research team will set up password protected Google Drive folders for each co-researcher to upload their photos and images. Access will be limited to Photovoice workshop facilitation staff who are members of the research team. Co-researchers will not have access to each other’s photos. Upon project completion, a digital photo album will be created for each contributing co-researcher and uploaded to their personal, encrypted folder.

### Knowledge translation

Following participation in the Photovoice workshop series, co-researchers will be thanked for their contributions and debriefed about what will happen with the information including knowledge translation activities (see below). Co-researchers may choose the degree to which they want to be involved in these activities. Co-researchers are the owners of the photos and accompanying text (Photovoice narratives) that they produce for the workshops. Each co-researcher will receive a personalized digital photo album containing their contributions in a PDF format which will be theirs to keep and which they may share if and where they so choose. To facilitate inclusive knowledge translation and exchange, linguistic translation of Photovoice captions will be supported by request into co-researchers’ (and their communities’) preferred language. Co-researchers will also receive e-communications with updates about the project and opportunities to stay involved (e.g., co-presenting at conferences, taking part in further arts-based activities and advocacy work, etc.).

***e-zine/virtual exhibit*:** Following the completion of each Photovoice workshop series, the caregiver co-researchers will be invited to co-develop a chapter for a Photovoice project e-zine. An e-zine is an electronic magazine that will be distributed in an electronic format, typically through a website or listserv with content that often includes images, text, and graphics [51]. The format of the e-zine works well for different purposes aligned with this project that include community building and information sharing. The e-zine will be developed using FlowPaper (a software program that enables the conversion of PDF documents into interactive web-based publications) and with the support of a research associate with experience in graphic design. Each chapter of the e-zine will be dedicated to highlighting Photovoice narratives and findings from one of the caregiver groups and will be hosted on a project website as a virtual exhibit with the consent of co-researchers. To facilitate the development of the e-zine, two virtual ‘office hours’ per group will be held at the end of each workshop series. During these sessions, co-researchers can meet individually or in small groups with the graphic designer to help co-design the e-zine chapter.

***Peer-reviewed manuscripts:*** Peer-reviewed publications will also form part of the study’s knowledge translation activities, with at least one group-specific manuscript anticipated from each of the four Photovoice workshop series. Co-researchers will be invited to participate in manuscript development as co-authors, and all co-researchers will have an opportunity to review and provide feedback on manuscripts arising from their workshop series before submission, regardless of whether they choose to take on a co-author role.

***Joint celebration event:*** Following the completion of the four Photovoice workshop series and e-zine development, we will bring together the co-researchers from all four groups, project team members, and CAB members for an in-person event to celebrate and share the work with one another. Transportation costs will be covered for all co-researchers and CAB members, as will caregiving costs. Food and drinks will be provided to support equitable access and engagement. The event will provide an opportunity for recognizing the work done thus far, for learning from one another, and for collaboratively discussing and prioritizing actionable ideas for policy and systems change.

***Strategic initiatives:*** Following the completion of the Photovoice workshops, findings will be shared with health system decision makers at the hospital where this project is situated, community service providers, local, municipal, provincial, and national leaders, etc. These partners actively advocate for support to caregivers and will help to explore pathways to drive evidence-based change.

## Discussion and conclusion

This protocol outlines a participatory, arts-informed study that will use Photovoice to amplify the lived experiences of caregivers from diverse communities in Peel Region, Ontario, Canada. The results of the Photovoice project will not only help to centre the strengths, needs, and perspectives of diverse caregivers across Peel Region, but will also contribute to the identification of ideas, practices, and innovations to better integrate caregivers in Ontario’s health and social care system. Moreover, by engaging in participatory research and knowledge mobilization initiatives, co-researchers in this this study will have opportunities to form bonds with other caregivers from across the region, develop a stronger sense of community, and build capacity in advocacy.

The findings and creative outputs from this project—most notably the co-created e-zine—will be shared with partners including caregivers, service providers, municipal leaders, and health system decision-makers in Peel, as well as with provincial and national partners such as the CCCE and the Ontario Caregiver Organization. These partners actively advocate for systems change for caregivers and will help explore pathways to further mobilize caregiver stories to increase awareness and advance change. The embedded knowledge translation and exchange approach within this study will involve the CAB in addition to hospital and community agency leaders. The purpose is to help realize change recommendations grounded in lived experiences highlighted by co-researchers during the Photovoice. Co-researchers themselves will engage in advocacy given their autonomy to promote their work via personal and community networks [52,53]**.** Potential next steps may include contributing to a local caregiving strategy that is specific to the context of Peel region while aligning with the CCCE’s national strategy [1]. In addition, this work will connect with future arts-based initiatives, such as a digital storytelling project, to further engage caregiver lived experience building upon the themed photovoice narratives collected in this first research phase.

While this project employs a rigorous participatory design, several limitations should be noted. For example, the study’s preference for English-speaking caregivers in Peel Region (unless otherwise requested), may restrict the applicability of findings to caregivers who are non-English speaking or who reside in different geographic settings. Additionally, while virtual Photovoice methods increase accessibility for many, they may also pose barriers for individuals with limited internet access, low digital literacy, or lack of privacy at home [44,46]. The time commitment required to participate in this study may also pose a barrier to caregivers engaged in multiple demanding responsibilities (e.g., caregiving, full- or part-time work, family care, etc.). Despite mitigation strategies (e.g., drop-in ‘office hours’, one-on-one tech support), the use of technology and time commitment may shape who is (and is not) able to participate and influence the findings [44,54]. Finally, while Photovoice offers valuable insight into lived experience, it is not designed to produce generalizable results [55]. Rather, the strength of this method lies in its depth and contextual richness, which can inform practice and policy recommendations in meaningful but non-universal ways.

## Supporting information

S1 File Co-researcher Photovoice Evaluation Discussion Guide. (DOCX)
