# Supplementary material for: Building capacity in health systems with and for caregivers: A photovoice protocol
Source: PLoS One. 2026 Sep 15;21(9):e0358550. doi: 10.1371/journal.pone.0358550 (PMC13577488; doi:10.1371/journal.pone.0358550)
Supplement: Supplementary file 1 [file pone.0358550.s001.docx]

**S1 File. Co-researcher Photovoice Evaluation Discussion Guide**

**Preamble:** Thank you for agreeing to participate in the feedback session. We are seeking your feedback on the process and to better understand your experience as a co-researcher participating in the photovoice workshops. We want to hear about what worked well, what did not work so well, and any ideas you may have about ways the photovoice process could be improved in the future.

There are no right or wrong answers to any of our questions; we are interested in your feedback and viewpoint. Participation in this feedback session is voluntary. You can withdraw from the study at any time and contact information to do so is provided in your study information and informed consent package.

We would like to audio-record the discussion so that we can fully capture all your insights and observations. After the discussion, the audio recording will be transcribed into written words and any names of individuals, including your name, and organizations will be removed. The written transcript will be used, with your permission, to help identify strengths and areas where we could improve our Photovoice process for co-researchers. We will removed identifying information and organize the feedback by themes or ideas and use these data to inform future workshops, and to write a paper reflecting on the process and identifying best practices for conducting photovoice workshops online.

We can stop recording or stop the discussion at any point, if you want, and you may decline to answer any question for any reason. Also, if you would like to take a break, please let me know. Do you have any questions before we begin? May I turn on the recorder?

**Hit START Button**

Semi-structured feedback discussion guide

(*Please note that this guide only represents the main themes to be discussed with co-researchers and as such does not include the various prompts that may also be used. Non- leading prompts will also be used, such as “Can you please tell me a little bit more about that?” and “What does that look like for you”. We will also ask for concrete examples of general descriptive statements that are made.)*

1. Please share a little about the experience of participating in the Photovoice workshops.
2. What was the most surprising or unexpected thing about participating in the Photovoice?
3. What aspects of the project did you enjoy or find meaningful?
4. What aspects of the project did you find challenging or frustrating?
5. What was it like sharing your photos and stories and hearing other co-researchers’ photos and stories during the workshops?
6. Looking back, is there anything you would have done differently in your participation?
7. What are some things with the project and workshop sessions that could have been done differently?
8. What do you see as opportunities to improve the process and your experience as a co-researcher during the photovoice workshops?
9. Is there anything else you would like to share about your experience in participating in the Photovoice that we did not get a chance to discuss?
