# Supplementary material for: Building capacity in health systems with and for caregivers: A photovoice protocol
Source: PLoS One. 2026 Sep 15;21(9):e0358550. doi: 10.1371/journal.pone.0358550 (PMC13577488; doi:10.1371/journal.pone.0358550)
Supplement: Supplementary file 2 [file pone.0358550.s003.docx]

**Author Response to Reviewer Comments**

**We would like to thank the reviewer for their thoughtful feedback, questions and insights on our Photovoice Protocol manuscript. We appreciate the opportunity to reflect on our work, and we believe that the manuscript has been improved as a result of their valuable comments and suggestions. We have responded to each enquiry or suggestion provided by the reviewer, below and in the revised manuscript. Please note that all page numbers referred to below correspond to the page numbers in the clean (not tracked changes) version of the revised manuscript.**

**Please note that we have also made a small number of additional minor revisions to the manuscript to reflect refinements made as the work has progressed. These changes are summarized at the end of this response letter.**

General Comments
**1. Sample distribution and cultural influence on findings: You mention that the sample reflects the population distribution of Pell’s residents, which is an important strength. However, given that the study’s findings will rely heavily on participants’ perceptions—often shaped by cultural norms, traditions, and community histories—I wonder how the demographic distribution (e.g., more than 50% South Asian participants, and a Black population twice the size of the Chinese population) might influence the results. Could this distribution affect the themes that emerge, and if so, how do you plan to account for or manage this in your analysis?**

**Thank you for raising this important point. We agree that participants’ social, cultural, and community contexts may shape how caregiving is experienced and understood and, consequently, the themes that emerge from the Photovoice discussions. We would like to clarify, however, that the demographic information presented in the manuscript describes the population of Peel Region and is not intended to suggest that the study sample will proportionally reproduce this distribution. Our purposive, maximum variation sampling approach is intended to include caregivers with a range of social identities and experiences rather than to achieve demographic representativeness. In the analysis, we will remain attentive to how co-researchers’ social locations and community contexts may contribute to similarities and differences in their experiences. At the same time, given the small number of co-researchers in each workshop series, the study is not designed to make systematic comparisons between ethnocultural or other groups or to attribute particular themes to culture *unless co-researchers themselves explicitly make those connections*. We have revised the manuscript to clarify the approach; specifically, we have made the following changes:**

- **In the Study Setting section (see page 6), we have added the following to the end of the paragraph:** Although maximum variation sampling will be used to recruit eligible caregivers with a range of identities and experiences, the final sample composition will depend on who expresses interest and is able to participate, and therefore may not proportionally reflect Peel Region’s population distribution.
- **In the Sampling and Recruitment section (see page 7), we have revised the first sentence to read as follows:** The sampling approach will be purposive and will use maximum variation sampling to support the inclusion of community members representing a diverse range of identities (i.e., age, gender, racialized group, ethnicity, religion, education) and experiences, rather than to achieve demographic representativeness.
- **In the Data Collection and Analysis section (see page 13), we have added the following:** Given the small, purposively selected groups, the analysis will not be designed to make systematic comparisons between ethnocultural groups or to generalize particular themes to these communities, although co-researchers may choose to explore similarities and differences across experiences if they arise organically during collaborative analysis.

**2. Knowledge translation activities: The manuscript mentions conference presentations as part of the knowledge translation plan, but it is unclear whether peer reviewed publications are also planned. Will articles be produced from this study, and will co researchers be included as co authors or contributors?**

**Thank you for pointing this out. Peer-reviewed publications are an important component of our knowledge translation plan. We anticipate developing at least one group-specific publication from each of the four Photovoice workshop series, and co-researchers will be invited to participate in manuscript development as co-authors. Regardless of whether they choose to take on a co-author role, all co-researchers will be offered an opportunity to review and provide feedback on manuscripts arising from their workshop series before submission.**

**We note that the manuscript already refers on page 5 to the recognition and inclusion of co-researchers in publications. To address your observation, we have revised this sentence to specify “including as co-authors” and have added the following brief paragraph to the knowledge translation section (see page 15) explicitly describing the planned peer-reviewed publications and opportunities for co-researcher involvement:**

***Peer-reviewed manuscripts:*** Peer-reviewed publications will also form part of the study’s knowledge translation activities, with at least one group-specific manuscript anticipated from each of the four Photovoice workshop series. Co-researchers will be invited to participate in manuscript development as co-authors, and all co-researchers will have an opportunity to review and provide feedback on manuscripts arising from their workshop series before submission, regardless of whether they choose to take on a co-author role.

**3. Ethical approval: While ethical considerations are addressed throughout the manuscript, it is not stated whether the study has been submitted to, or approved by, a research ethics committee. Please clarify the status of ethics approval.**

**Thank you for identifying this omission. At the time of the initial manuscript submission, the research ethics application was still under review. The study has since received approval from the Trillium Health Partners Research Ethics Board (REB study ID: #1258). We have added an Ethics approval subsection to the Materials and methods section to clarify the study’s current approval status (see page 6).**
 **Specific Comments by Page**
**Page 5
• Spell out Community Based Participatory Research (CBPR) at first mention~~.~~**

**Thank you for spotting this omission. We have spelled it out at the first mention (on page 5).**

**• At the end of the last paragraph: What are the characteristics of the individuals sitting on the Community Advisory Board (CAB)?**

**Thank you for requesting further information about the composition of the Community Advisory Board. The CAB includes current and former caregivers with experience supporting children, parents, spouses, friends, and older adults across a range of health and social care contexts, including cancer, dementia, autism, Parkinson’s disease, mental health, and addictions. The CAB includes members from different racial and ethnocultural communities, and members’ lived, community and professional experiences are grounded in the Peel Region context. Standardized sociodemographic information was not collected from CAB members, so we are unable to provide a formal demographic breakdown. We have revised the manuscript to describe the breadth of members’ caregiving, community, professional, and cultural perspectives while avoiding claims of demographic representativeness. The revised portion of the paragraph in question (see page 5) now reads as follows:**

In September of 2024, a CAB was formed comprising current and former caregivers with experience supporting children, parents, spouses, friends, and older adults across a range of health and social care contexts. The CAB includes members from different racial and ethnocultural communities, as well as individuals with experience in caregiver and patient advisory roles, community services, disability advocacy, home care, seniors’ services, research, and health-system leadership. Members’ lived, community, and professional experiences are grounded in the Peel Region context, enabling them to provide locally relevant guidance throughout the Photovoice project.

**Page 6
• First paragraph: Please clarify what qualifies someone as a “recent immigrant” in this context.**

**Thank you for requesting clarification. We have updated this section using more recent 2021 Census data. In the 2021 Census, “recent immigrants” refers to individuals who first obtained landed immigrant or permanent resident status between 2016 and 2021; recent immigrants comprised approximately 14% of Peel Region’s immigrant population.**

**In updating the section, we also identified an error in the original description of Peel’s South Asian population and have corrected this by providing a more accurate description of the racialized population in Peel.**

**The revised passage (see page 6) now reads as follows:**

In 2021, approximately 69% of Peel residents belonged to racialized population groups group, with South Asian residents comprising the largest racialized population in the region, followed by Black, Filipino, Chinese, and Arab residents. Recent immigrants—defined as immigrants who first obtained landed immigrant or permanent resident status between January 1, 2016 and May 11, 2021—comprised approximately 14.0% of Peel’s immigrant population.

**• Study participants: Define what “complex care” entails for “caregivers of children with complex care needs.”**

**Thank you for noting the need for a clearer definition. We have revised the Study participants section to provide more specific eligibility criteria for the group of caregivers of children with complex care needs. The eligibility criteria for this group now reads as follows in the revised manuscript (see page 7):**

**Group 1 criteria** (***caregivers of children with complex care needs):*** Have experience as an unpaid parent, family member, friend, or other support for a child aged 0-18 years with a diagnosed or suspected long-term developmental or behavioural condition, such as autism spectrum disorder, developmental delay, or behavioural challenges that affect daily activities. The child must experience noticeable challenges in everyday functioning compared with peers of a similar age, require ongoing healthcare, therapeutic, community, or school-based services or supports (whether or not these are currently available), and require a level of care coordination or advocacy that places significant demands on the family.

**• There is inconsistency between “caregivers OF children…” and “caregivers FOR children…” across sections. Please harmonize the phrasing.**

**Thank you for identifying this inconsistency. We have revised the wording throughout so that we consistently refer to “caregivers OF…”.**

**Page 7
• Second paragraph: Provide a brief explanation of the “maximum variation sampling approach” or include a reference.**

**Thank you for this suggestion. We have revised the Sampling and recruitment section to clarify that maximum variation sampling involves purposively recruiting co-researchers with a range of identities and caregiving experiences to capture diverse perspectives within each workshop series. The revised section now reads as follows (see page 7):**

During an initial telephone screening conversation, the researchers will assess eligibility and co-researcher attributes to support a maximum variation sampling approach, which involves purposively seeking co-researchers with a range of identities and caregiving experiences to capture diverse perspectives within each workshop series.

**• Third paragraph: Specify the types of organizations that will be involved.**

**Thank you for this suggestion. We have revised the Sampling and recruitment section to specify the types of organizations that may support recruitment, and the revised section now reads as follows (see pages 7-8):**

Electronic flyers will be distributed to collaborators for dissemination through email distribution lists and posting at recommended sites and on partners’ social media platforms. These collaborators may include caregiver support and advocacy organizations, community health and social service organizations, hospital-based programs and clinics, patient and family advisory groups, and organizations serving children with complex care needs, older adults, young adults with mental health challenges, and women in multigenerational caregiving roles (i.e., sandwich generation caregivers).

**Page 8
• First paragraph: The timeline indicates recruitment beginning in September 2025 and workshops starting in October 2025. Given the current date, this may need updating. Have these activities already taken place?**

**Thank you for raising this point. At the time of revision, two of the four Photovoice workshop series have been completed and a third is underway. However, because this manuscript presents the study protocol, we have retained the prospective description of the planned methods rather than revising the manuscript to report study progress or findings. To avoid confusion arising from dates that have now passed, we have revised the manuscript to describe the anticipated sequencing and duration of the workshop series without specifying the original recruitment and workshop start dates. The revised passage in the manuscript (see page 8) now reads as follows:**

The four workshop series will be conducted sequentially over approximately twelve months. Data collection and collaborative theming will be completed for each group before the findings are brought together through the planned cross-group knowledge translation activities.

**• Third paragraph:
o Regarding “co researchers may access online public domain images”: Will the research team provide these images or guidance on accessing them? I am concerned about the copyright issues.**

**Thank you for raising this concern. We have revised the manuscript to clarify that co-researchers will receive guidance on the appropriate use of online images and that copyright and reuse permissions will be addressed as part of the Photovoice training. The option to use online images is intended as a lower-barrier starting point for co-researchers who may initially feel hesitant to take or share their own photographs, particularly when reflecting on sensitive or stigmatized experiences. In this context, an online image may serve as a catalyst for reflection and discussion, with co-researchers supported to move toward original photographs or artwork if and when they feel comfortable doing so. Only images with appropriate permissions will be used in public-facing outputs. The revised passage in the manuscript (see page 9) now reads as follows:**

Alternatively, co-researchers may access online images that are clearly identified as being in the public domain or otherwise licensed for reuse. This option may provide a lower-barrier starting point for co-researchers who are initially hesitant to take or share personal photographs, including when reflecting on sensitive or stigmatized experiences. The research team will provide guidance on locating appropriate images and documenting their source and licensing information. Any image for which reuse permissions cannot be verified will not be included in public-facing knowledge translation materials; co-researchers will instead be supported to replace it with an original photograph or artwork.

**o Why don’t you consider including past oriented prompts to explore how participants perceive changes over time.**

**Thank you for this suggestion. The experiential prompt is intentionally broad and may encompass past as well as current caregiving experiences, including participants’ perceptions of how their experiences have changed over time. We have retained the future-oriented prompt because envisioning what a society that values and supports caregivers could look like aligns closely with Photovoice’s action and advocacy orientation and supports the development of recommendations for policy and systems change. As the prompts will be co-created and refined with each group, co-researchers may also choose to incorporate more explicit reflection on changes over time where this is important to them.**

**o Clarify whether Workshops 1 and 2 occur within the same week. It is clearer for the other workshops.**

**Thank you for noting this. We have clarified in Table 1 that there will be at least one week between Workshops 1 and 2. (See page 10)**

**Page 11
• Last sentence: “Photovoice co facilitators will be available for meetings via Zoom, phone, or email.” As written, it suggests that meetings may occur via email. Consider rephrasing for clarity.**

**Thank you for noting this ambiguity. We have revised the sentence (see page 12) as follows:**

Additionally, Photovoice co-facilitators will be available to meet with co-researchers via Zoom or phone and to provide support by email.

**Page 12
• In “Phase 2: Photovoice evaluation,” the text refers to four Photovoice workshop series, whereas the abstract and Table 1 mention six workshops. Please clarify this discrepancy.**

**Thank you for identifying this potential ambiguity. The study includes four distinct Photovoice workshop series, one for each caregiver group, and each series will consist of approximately six workshops. We have revised the Phase 2 description to make this distinction explicit. The revised description (see page 12) reads as follows:**

Following the completion of the four Photovoice workshop series, one for each caregiver group and each consisting of approximately six workshops (see Table 1), co-researchers across all groups (up to 20 total, or 5 from each group) will be invited to participate in feedback interviews or focus groups.

**Page 14
• The end of the second paragraph in the Discussion and Conclusion suggests that this is the first phase of a larger research program. If this is the case, it would be helpful to state this clearly earlier in the manuscript, particularly when outlining the study objectives.**

**Thank you for noting this ambiguity. We have revised the Study objectives section (as well as the relevant sections of the Abstract; see page 2) to clarify that the Photovoice study described in this protocol is a foundational component of a broader four-year research program. We have also clarified that the listed objectives are the overarching objectives of the broader program and distinguished these from the specific scope of this protocol. The revised section (see page 4) now reads as follows:**

**Study objectives**

The Photovoice study outlined in this protocol is a foundational component of a broader four-year research program being conducted in collaboration with the CCCE. The broader program aims to advance caregiver-informed health-system priorities and establish a Research and Innovation Hub focused on caregivers’ essential role in health and social systems. The overarching objectives of the broader program are to: 1) co-design policy and practice recommendations by understanding the lived experiences (and user-centred priorities) of caregivers from diverse communities and health contexts; 2) mobilize evidence and build lived expertise knowledge to help communicate and amplify caregiver voices for policy, system, and practice change; 3) co-design an implementation toolkit to support health and social systems in adopting best practices for caregivers; and 4) establish a Research and Innovation Hub focused on caregivers’ essential role in health and social systems.

This protocol specifically outlines plans for a Photovoice workshop series and related knowledge translation activities. Photovoice is an inclusive and participatory arts-informed method (22-24) that allows for lived experience to be explored, better understood, and leveraged in advocacy work. The research team will work alongside caregivers from diverse communities to co-create a Photovoice gallery and collaborative themed analysis that highlights promising practices, challenges, and priorities for advocacy and systems change (22, 23). The evidence, recommendations, and knowledge translation outputs generated through the Photovoice activities will contribute to the overarching objectives of the broader research program.

***Minor additional revisions made:***

**• We have made the following changes to language and eligibility for the caregiver groups in the following workshop series (in addition to changes for group #1, as already described above in the response to the reviewer):**

**-Group 3 is now described throughout the manuscript as “caregivers supporting young adults with mental health challenges” (previously described as caregivers supporting individuals with mental wellness concerns).**

**-Group 4 eligibility now reads as follows (see page 7): “1) self-identify as a woman; 2) be a caregiver for at least two generations simultaneously, with at least one of those individuals having a disability, medical condition, or illness.” Previously, the manuscript wording could be interpreted as requiring *all* individuals being cared for across generations to have a disability, medical condition, or illness. The revised wording clarifies that this criterion applies to at least one of the individuals receiving care.**

**• We have revised the manuscript to reflect that NVivo, rather than MAXQDA, will be used for qualitative data management and analysis (see page 14).**

**• Table 1, Page 12: We have added “electronic magazine” in brackets next to the first mention of “e-zine” to improve clarity.**
