# Supplementary material for: Building capacity in health systems with and for caregivers: A photovoice protocol
Source: PLoS One. 2026 Sep 15;21(9):e0358550. doi: 10.1371/journal.pone.0358550 (PMC13577488; doi:10.1371/journal.pone.0358550)
Supplement: Peer review history

# Peer review: Decision Letter 0

**DOI:** 10.1371/journal.pone.0358550.r001

**Authors:** Ilse Bloom

24 Jul 2026

Dear Dr. Fuentes,

Thank you for submitting your manuscript to PLOS ONE. After careful consideration, we feel that it has merit but does not fully meet PLOS ONE’s publication criteria as it currently stands. Therefore, we invite you to submit a revised version of the manuscript that addresses the points raised during the review process.

Please note that we have only been able to secure a single reviewer to assess your manuscript. We are issuing a decision on your manuscript at this point to prevent further delays in the evaluation of your manuscript. Please be aware that the editor who handles your revised manuscript might find it necessary to invite additional reviewers to assess this work once the revised manuscript is submitted. However, we will aim to proceed on the basis of this single review if possible.

The comments from the reviewer can be found below. Please review these comments and make the appropriate revisions to your manuscript alongside a point-by-point response to the concerns raised.

Please submit your revised manuscript by Sep 06 2026 11:59PM. If you will need more time than this to complete your revisions, please reply to this message or contact the journal office at plosone@plos.org. When you're ready to submit your revision, log on to https://www.editorialmanager.com/pone/ and select the 'Submissions Needing Revision' folder to locate your manuscript file.

- A letter that responds to each point raised by the academic editor and reviewer(s). You should upload this letter as a separate file labeled 'Response to Reviewers'.
- A marked-up copy of your manuscript that highlights changes made to the original version. You should upload this as a separate file labeled 'Revised Manuscript with Track Changes'.
- An unmarked version of your revised paper without tracked changes. You should upload this as a separate file labeled 'Manuscript'.

If you would like to make changes to your financial disclosure, please include your updated statement in your cover letter. Guidelines for resubmitting your figure files are available below the reviewer comments at the end of this letter.

If applicable, we recommend that you deposit your laboratory protocols in protocols.io to enhance the reproducibility of your results. Protocols.io assigns your protocol its own identifier (DOI) so that it can be cited independently in the future. For instructions see: https://journals.plos.org/plosone/s/submission-guidelines#loc-laboratory-protocols. Additionally, PLOS ONE offers an option for publishing peer-reviewed Lab Protocol articles, which describe protocols hosted on protocols.io. Read more information on sharing protocols at https://plos.org/protocols?utm_medium=editorial-email&utm_source=authorletters&utm_campaign=protocols.

As the corresponding author, your ORCID iD is verified in the submission system and will appear in the published article. PLOS supports the use of ORCID, and we encourage all coauthors to register for an ORCID iD and use it as well. Please encourage your coauthors to verify their ORCID iD within the submission system before final acceptance, as unverified ORCID iDs will not appear in the published article. *Only*  the individual author can complete the verification step; PLOS staff *cannot*  verify ORCID iDs on behalf of authors.

We look forward to receiving your revised manuscript.

Kind regards,

Ilse Bloom

Staff Editor

PLOS One

Journal Requirements:

When submitting your revision, we need you to address these additional requirements.

1. Please ensure that your manuscript meets PLOS ONE's style requirements, including those for file naming. The PLOS ONE style templates can be found at

https://journals.plos.org/plosone/s/file?id=wjVg/PLOSOne_formatting_sample_main_body.pdf and

https://journals.plos.org/plosone/s/file?id=ba62/PLOSOne_formatting_sample_title_authors_affiliations.pdf

2. When completing the data availability statement of the submission form, you indicated that you will make your data available on acceptance. We strongly recommend all authors decide on a data sharing plan before acceptance, as the process can be lengthy and hold up publication timelines. Please note that, though access restrictions are acceptable now, your entire data will need to be made freely accessible if your manuscript is accepted for publication. This policy applies to all data except where public deposition would breach compliance with the protocol approved by your research ethics board. If you are unable to adhere to our open data policy, please kindly revise your statement to explain your reasoning and we will seek the editor's input on an exemption. Please be assured that, once you have provided your new statement, the assessment of your exemption will not hold up the peer review process.

3. Please include your full ethics statement in the ‘Methods’ section of your manuscript file. In your statement, please include the full name of the IRB or ethics committee who approved or waived your study, as well as whether or not you obtained informed written or verbal consent. If consent was waived for your study, please include this information in your statement as well.

4. Please include captions for your Supporting Information files at the end of your manuscript, and update any in-text citations to match accordingly. Please see our Supporting Information guidelines for more information: http://journals.plos.org/plosone/s/supporting-information.

5. If the reviewer comments include a recommendation to cite specific previously published works, please review and evaluate these publications to determine whether they are relevant and should be cited. There is no requirement to cite these works unless the editor has indicated otherwise.

[Note: HTML markup is below. Please do not edit.]

Reviewers' comments:

Reviewer's Responses to Questions

**Comments to the Author**

1. Does the manuscript provide a valid rationale for the proposed study, with clearly identified and justified research questions?

Reviewer #1: Yes

**********

2. Is the protocol technically sound and planned in a manner that will lead to a meaningful outcome and allow testing the stated hypotheses?

Reviewer #1: Yes

**********

3. Is the methodology feasible and described in sufficient detail to allow the work to be replicable?

Reviewer #1: Yes

**********

4. Have the authors described where all data underlying the findings will be made available when the study is complete?

The PLOS Data policy

Reviewer #1: Yes

**********

5. Is the manuscript presented in an intelligible fashion and written in standard English?

Reviewer #1: Yes

**********

Please use the space provided to explain your answers to the questions above and, if applicable, provide comments about issues authors must address before this protocol can be accepted for publication. You may also include additional comments for the author, including concerns about research or publication ethics.

You may also provide optional suggestions and comments to authors that they might find helpful in planning their study.

Reviewer #1: Thank you for the opportunity to review this manuscript.

The topic addressed in this study is highly relevant and focuses on populations that are often underserved or overlooked. The use of a community based participatory research (CBPR) approach is particularly commendable, especially the meaningful involvement of participants as co researchers. The choice of Photovoice is also appropriate, as it allows the research team to capture not only observable realities but also the perspectives, emotions, and lived experiences of participants on a sensitive and important issue.

The methodology is clearly articulated, and the detailed procedural descriptions greatly support the reader’s understanding of the project. The bibliography is strong and provides solid support for the conceptual and methodological choices made throughout the manuscript.

I do, however, have several questions and suggestions for clarification that I believe will strengthen the manuscript.

General Comments

1. Sample distribution and cultural influence on findings You mention that the sample reflects the population distribution of Pell’s residents, which is an important strength. However, given that the study’s findings will rely heavily on participants’ perceptions—often shaped by cultural norms, traditions, and community histories—I wonder how the demographic distribution (e.g., more than 50% South Asian participants, and a Black population twice the size of the Chinese population) might influence the results. Could this distribution affect the themes that emerge, and if so, how do you plan to account for or manage this in your analysis?

2. Knowledge translation activities The manuscript mentions conference presentations as part of the knowledge translation plan, but it is unclear whether peer reviewed publications are also planned. Will articles be produced from this study, and will co researchers be included as co authors or contributors?

3. Ethical approval While ethical considerations are addressed throughout the manuscript, it is not stated whether the study has been submitted to, or approved by, a research ethics committee. Please clarify the status of ethics approval.

Specific Comments by Page

Page 5

• Spell out Community Based Participatory Research (CBPR) at first mention.

• At the end of the last paragraph: What are the characteristics of the individuals sitting on the Community Advisory Board (CAB)?

Page 6

• First paragraph: Please clarify what qualifies someone as a “recent immigrant” in this context.

• Study participants: Define what “complex care” entails for “caregivers of children with complex care needs.”

• There is inconsistency between “caregivers OF children…” and “caregivers FOR children…” across sections. Please harmonize the phrasing.

Page 7

• Second paragraph: Provide a brief explanation of the “maximum variation sampling approach” or include a reference.

• Third paragraph: Specify the types of organizations that will be involved.

Page 8

• First paragraph: The timeline indicates recruitment beginning in September 2025 and workshops starting in October 2025. Given the current date, this may need updating. Have these activities already taken place?

• Third paragraph:

o Regarding “co researchers may access online public domain images”: Will the research team provide these images or guidance on accessing them? I am concerned about the copyright issues.

o Why don’t you consider including past oriented prompts to explore how participants perceive changes over time.

o Clarify whether Workshops 1 and 2 occur within the same week. It is clearer for the other workshops.

Page 11

• Last sentence: “Photovoice co facilitators will be available for meetings via Zoom, phone, or email.” As written, it suggests that meetings may occur via email. Consider rephrasing for clarity.

Page 12

• In “Phase 2: Photovoice evaluation,” the text refers to four Photovoice workshop series, whereas the abstract and Table 1 mention six workshops. Please clarify this discrepancy.

Page 14

• The end of the second paragraph in the Discussion and Conclusion suggests that this is the first phase of a larger research program. If this is the case, it would be helpful to state this clearly earlier in the manuscript, particularly when outlining the study objectives.

**********

what does this mean?). If published, this will include your full peer review and any attached files.

If you choose “no”, your identity will remain anonymous but your review may still be made public.

**Do you want your identity to be public for this peer review?**  For information about this choice, including consent withdrawal, please see our Privacy Policy

Reviewer #1: **Yes:** Charles Daboné

**********

[NOTE: If reviewer comments were submitted as an attachment file, they will be attached to this email and accessible via the submission site. Please log into your account, locate the manuscript record, and check for the action link "View Attachments". If this link does not appear, there are no attachment files.]

To ensure your figures meet our technical requirements, please review our figure guidelines: https://journals.plos.org/plosone/s/figures

You may also use PLOS’s free figure tool, NAAS, to help you prepare publication quality figures: https://journals.plos.org/plosone/s/figures#loc-tools-for-figure-preparation.

NAAS will assess whether your figures meet our technical requirements by comparing each figure against our figure specifications.

# Author comment: Author response to Decision Letter 1

**DOI:** 10.1371/journal.pone.0358550.r002

21 Aug 2026

(The Response to Reviewers is included below as well as in the attached full revision submission document).

Author Response to Reviewer Comments

We would like to thank the reviewer for their thoughtful feedback, questions and insights on our Photovoice Protocol manuscript. We appreciate the opportunity to reflect on our work, and we believe that the manuscript has been improved as a result of their valuable comments and suggestions. We have responded to each enquiry or suggestion provided by the reviewer, below and in the revised manuscript. Please note that all page numbers referred to below correspond to the page numbers in the clean (not tracked changes) version of the revised manuscript.

Please note that we have also made a small number of additional minor revisions to the manuscript to reflect refinements made as the work has progressed. These changes are summarized at the end of this response letter.

General Comments

1. Sample distribution and cultural influence on findings: You mention that the sample reflects the population distribution of Pell’s residents, which is an important strength. However, given that the study’s findings will rely heavily on participants’ perceptions—often shaped by cultural norms, traditions, and community histories—I wonder how the demographic distribution (e.g., more than 50% South Asian participants, and a Black population twice the size of the Chinese population) might influence the results. Could this distribution affect the themes that emerge, and if so, how do you plan to account for or manage this in your analysis?

Thank you for raising this important point. We agree that participants’ social, cultural, and community contexts may shape how caregiving is experienced and understood and, consequently, the themes that emerge from the Photovoice discussions. We would like to clarify, however, that the demographic information presented in the manuscript describes the population of Peel Region and is not intended to suggest that the study sample will proportionally reproduce this distribution. Our purposive, maximum variation sampling approach is intended to include caregivers with a range of social identities and experiences rather than to achieve demographic representativeness. In the analysis, we will remain attentive to how co-researchers’ social locations and community contexts may contribute to similarities and differences in their experiences. At the same time, given the small number of co-researchers in each workshop series, the study is not designed to make systematic comparisons between ethnocultural or other groups or to attribute particular themes to culture unless co-researchers themselves explicitly make those connections. We have revised the manuscript to clarify the approach; specifically, we have made the following changes:

- In the Study Setting section (see page 6), we have added the following to the end of the paragraph: Although maximum variation sampling will be used to recruit eligible caregivers with a range of identities and experiences, the final sample composition will depend on who expresses interest and is able to participate, and therefore may not proportionally reflect Peel Region’s population distribution.

- In the Sampling and Recruitment section (see page 7), we have revised the first sentence to read as follows: The sampling approach will be purposive and will use maximum variation sampling to support the inclusion of community members representing a diverse range of identities (i.e., age, gender, racialized group, ethnicity, religion, education) and experiences, rather than to achieve demographic representativeness.

- In the Data Collection and Analysis section (see page 13), we have added the following: Given the small, purposively selected groups, the analysis will not be designed to make systematic comparisons between ethnocultural groups or to generalize particular themes to these communities, although co-researchers may choose to explore similarities and differences across experiences if they arise organically during collaborative analysis.

2. Knowledge translation activities: The manuscript mentions conference presentations as part of the knowledge translation plan, but it is unclear whether peer reviewed publications are also planned. Will articles be produced from this study, and will co researchers be included as co authors or contributors?

Thank you for pointing this out. Peer-reviewed publications are an important component of our knowledge translation plan. We anticipate developing at least one group-specific publication from each of the four Photovoice workshop series, and co-researchers will be invited to participate in manuscript development as co-authors. Regardless of whether they choose to take on a co-author role, all co-researchers will be offered an opportunity to review and provide feedback on manuscripts arising from their workshop series before submission.

We note that the manuscript already refers on page 5 to the recognition and inclusion of co-researchers in publications. To address your observation, we have revised this sentence to specify “including as co-authors” and have added the following brief paragraph to the knowledge translation section (see page 15) explicitly describing the planned peer-reviewed publications and opportunities for co-researcher involvement:

Peer-reviewed manuscripts: Peer-reviewed publications will also form part of the study’s knowledge translation activities, with at least one group-specific manuscript anticipated from each of the four Photovoice workshop series. Co-researchers will be invited to participate in manuscript development as co-authors, and all co-researchers will have an opportunity to review and provide feedback on manuscripts arising from their workshop series before submission, regardless of whether they choose to take on a co-author role.

3. Ethical approval: While ethical considerations are addressed throughout the manuscript, it is not stated whether the study has been submitted to, or approved by, a research ethics committee. Please clarify the status of ethics approval.

Thank you for identifying this omission. At the time of the initial manuscript submission, the research ethics application was still under review. The study has since received approval from the Trillium Health Partners Research Ethics Board (REB study ID: #1258). We have added an Ethics approval subsection to the Materials and methods section to clarify the study’s current approval status (see page 6).

Specific Comments by Page

Page 5

• Spell out Community Based Participatory Research (CBPR) at first mention.

Thank you for spotting this omission. We have spelled it out at the first mention (on page 5).

• At the end of the last paragraph: What are the characteristics of the individuals sitting on the Community Advisory Board (CAB)?

Thank you for requesting further information about the composition of the Community Advisory Board. The CAB includes current and former caregivers with experience supporting children, parents, spouses, friends, and older adults across a range of health and social care contexts, including cancer, dementia, autism, Parkinson’s disease, mental health, and addictions. The CAB includes members from different racial and ethnocultural communities, and members’ lived, community and professional experiences are grounded in the Peel Region context. Standardized sociodemographic information was not collected from CAB members, so we are unable to provide a formal demographic breakdown. We have revised the manuscript to describe the breadth of members’ caregiving, community, professional, and cultural perspectives while avoiding claims of demographic representativeness. The revised portion of the paragraph in question (see page 5) now reads as follows:

In September of 2024, a CAB was formed comprising current and former caregivers with experience supporting children, parents, spouses, friends, and older adults across a range of health and social care contexts. The CAB includes members from different racial and ethnocultural communities, as well as individuals with experience in caregiver and patient advisory roles, community services, disability advocacy, home care, seniors’ services, research, and health-system leadership. Members’ lived, community, and professional experiences are grounded in the Peel Region context, enabling them to provide locally relevant guidance throughout the Photovoice project.

Page 6

• First paragraph: Please clarify what qualifies someone as a “recent immigrant” in this context.

Thank you for requesting clarification. We have updated this section using more recent 2021 Census data. In the 2021 Census, “recent immigrants” refers to individuals who first obtained landed immigrant or permanent resident status between 2016 and 2021; recent immigrants comprised approximately 14% of Peel Region’s immigrant population.

In updating the section, we also identified an error in the original description of Peel’s South Asian population and have corrected this by providing a more accurate description of the racialized population in Peel.

The revised passage (see page 6) now reads as follows:

In 2021, approximately 69% of Peel residents belonged to racialized population groups group, with South Asian residents comprising the largest racialized population in the region, followed by Black, Filipino, Chinese, and Arab residents. Recent immigrants—defined as immigrants who first obtained landed immigrant or permanent resident status between January 1, 2016 and May 11, 2021—comprised approximately 14.0% of Peel’s immigrant population.

• Study participants: Define what “complex care” entails for “caregivers of children with complex care needs.”

Thank you for noting the need for a clearer definition. We have revised the Study participants section to provide more specific eligibility criteria for the group of caregivers of children with complex care needs. The eligibility criteria for this group now reads as follows in the revised manuscript (see page 7):

Group 1 criteria (caregivers of children with complex care needs): Have experience as an unpaid parent, family member, friend, or other support for a child aged 0-18 years with a diagnosed or suspected long-term developmental or behavioural condition, such as autism spectrum disorder, developmental delay, or behavioural challenges that affect daily activities. The child must experience noticeable challenges in everyday functioning compared with peers of a similar age, require ongoing healthcare, therapeutic, community, or school-based services or supports (whether or not these are currently available), and require a level of care coordination or advocacy that places significant demands on the family.

• There is inconsistency between “caregivers OF children…” and “caregivers FOR children…” across sections. Please harmonize the phrasing.

Thank you for identifying this inconsistency. We have revised the wording throughout so that we consistently refer to “caregivers OF…”.

Page 7

• Second paragraph: Provide a brief explanation of the “maximum variation sampling approach” or include a reference.

Thank you for this suggestion. We have revised the Sampling and recruitment section to clarify that maximum variation sampling involves purposively recruiting co-researchers with a range of identities and caregiving experiences to capture diverse perspectives within each workshop series. The revised section now reads as follows (see page 7):

During an initial telephone screening conversation, the researchers will assess eligibility and co-researcher attributes to support a maximum variation sampling approach, which involves purposively seeking co-researchers with a range of identities and caregiving experiences to capture diverse perspectives within each workshop series.

• Third paragraph: Specify the types of organizations that will be involved.

Thank you for this suggestion. We have revised the Sampling and recruitment section to specify the types of organizations that may support recruitment, and the revised section now reads as follows (see pages 7-8):

Electronic flyers will be distributed to collaborators for dissemination through email distribution lists and posting at recommended sites and on partners’ social media platforms. These collaborators may include caregiver support and advocacy organizations, community health and social service organizations, hospital-based programs and clinics, patient and family advisory groups, and organizations serving children with complex care needs, older adults, young adults with mental health challenges, and women in multigenerational caregiving roles (i.e., sandwich generation caregivers).

Page 8

• First paragraph: The timeline indicates recruitment beginning in September 2025 and workshops starting in October 2025. Given the current date, this may need updating. Have these activities already taken place?

Thank you for raising this point. At the time of revision, two of the four Photovoice workshop series have been completed and a third is underway. However, because this manuscript presents the study protocol, we have retained the prospective description of the planned methods rather than revising the manuscript to report study progress or findings. To avoid confusion arising from dates that have now passed, we have revised the manuscript to describe the anticipated sequencing and duration of the workshop series without specifying the original recruitment and workshop start dates. The revised passage in the manuscript (see page 8) now reads as follows:

The four workshop series will be conducted sequentially over approximately twelve months. Data collection and collaborative theming will be completed for each group before the findings are brought together through the planned cross-group knowledge translation activities.

• Third paragraph:

o Regarding “co researchers may access online public domain images”: Will the research team provide these images or guidance on accessing them? I am concerned about the copyright issues.

Thank you for raising this concern. We have revised the manuscript to clarify that co-researchers will receive guidance on the appropriate use of online images and that copyright and reuse permissions will be addressed as part of the Photovoice training. The option to use online images is intended as a lower-barrier starting point for co-researchers who may initially feel hesitant to take or share their own photographs, particularly when reflecting on sensitive or stigmatized experiences. In this context, an online image may serve as a catalyst for reflection and discussion, with co-researchers supported to move toward original photographs or artwork if and when they feel comfortable doing so. Only images with appropriate permissions will be used in public-facing outputs. The revised passage in the manuscript (see page 9) now reads as follows:

Alternatively, co-researchers may access online images that are clearly identified as being in the public domain or otherwise licensed for reuse. This option may provide a lower-barrier starting point for co-researchers who are initially hesitant to take or share personal photographs, including when reflecting on sensitive or stigmatized experiences. The research team will provide guidance on locating appropriate images and documenting their source and licensing information. Any image for which reuse permissions cannot be verified will not be included in public-facing knowledge translation materials; co-researchers will instead be supported to replace it with an original photograph or artwork.

o Why don’t you consider including past oriented prompts to explore how participants perceive changes over time.

Thank you for this suggestion. The experiential prompt is intentionally broad and may encompass past as well as current caregiving experiences, including participants’ perceptions of how their experiences have changed over time. We have retained the future-oriented prompt because envisioning what a society that values and supports caregivers could look like aligns closely with Photovoice’s action and advocacy orientation and supports the development of recommendations for policy and systems change. As the prom

Attachment Submitted filename: Response to Reviewers.docx

# Editor report: Decision Letter 1

**DOI:** 10.1371/journal.pone.0358550.r003

**Authors:** Jennifer Annette Campbell

3 Sep 2026

Building Capacity in Health Systems with and for Caregivers: A Photovoice protocol

PONE-D-25-48467R1

Dear Dr. Fuentes,

We’re pleased to inform you that your manuscript has been judged scientifically suitable for publication and will be formally accepted for publication once it meets all outstanding technical requirements.

Within one week, you’ll receive an e-mail detailing the required amendments. When these have been addressed, you’ll receive a formal acceptance letter and your manuscript will be scheduled for publication.

An invoice will be generated when your article is formally accepted. Please note, if your institution has a publishing partnership with PLOS and your article meets the relevant criteria, all or part of your publication costs will be covered. Please make sure your user information is up-to-date by logging into Editorial Manager at Editorial Manager® and clicking the ‘Update My Information' link at the top of the page. For questions related to billing, please contact billing support.

If your institution or institutions have a press office, please notify them about your upcoming paper to help maximize its impact. If they’ll be preparing press materials, please inform our press team as soon as possible -- no later than 48 hours after receiving the formal acceptance. Your manuscript will remain under strict press embargo until 2 pm Eastern Time on the date of publication. For more information, please contact onepress@plos.org.

Kind regards,

Jennifer Annette Campbell

Academic Editor

PLOS One

Additional Editor Comments (optional):

Reviewers' comments:

# Editor report: Acceptance letter

**DOI:** 10.1371/journal.pone.0358550.r004

**Authors:** Jennifer Annette Campbell

PONE-D-25-48467R1

PLOS One

Dear Dr. Fuentes,

I'm pleased to inform you that your manuscript has been deemed suitable for publication in PLOS One. Congratulations! Your manuscript is now being handed over to our production team.

At this stage, our production department will prepare your paper for publication. This includes ensuring the following:

* All references, tables, and figures are properly cited

* All relevant supporting information is included in the manuscript submission,

* There are no issues that prevent the paper from being properly typeset

You will receive further instructions from the production team, including instructions on how to review your proof when it is ready. Please keep in mind that we are working through a large volume of accepted articles, so please give us a few days to review your paper and let you know the next and final steps.

Lastly, if your institution or institutions have a press office, please let them know about your upcoming paper now to help maximize its impact. If they'll be preparing press materials, please inform our press team within the next 48 hours. Your manuscript will remain under strict press embargo until 2 pm Eastern Time on the date of publication. For more information, please contact onepress@plos.org.

You will receive an invoice from PLOS for your publication fee after your manuscript has reached the completed accept phase. If you receive an email requesting payment before acceptance or for any other service, this may be a phishing scheme. Learn how to identify phishing emails and protect your accounts at https://explore.plos.org/phishing.

If we can help with anything else, please email us at customercare@plos.org.

Thank you for submitting your work to PLOS One and supporting open access.

Kind regards,

PLOS One Editorial Office Staff

on behalf of

Dr. Jennifer Annette Campbell

Academic Editor

PLOS One
